# The Effect of Sub-Concussive Impacts during a Rugby Tackling Drill on Brain Function

**DOI:** 10.3390/brainsci10120960

**Published:** 2020-12-10

**Authors:** Colm McNabb, Tahere Reha, Julia Georgieva, Angela Jacques, Kevin Netto, Andrew P. Lavender

**Affiliations:** 1 School of Physiotherapy and Exercise Science, Curtin University, Bentley 6102, Australia; colm.mcnabb@student.curtin.edu.au (C.M.); tahere.reha@student.curtin.edu.au (T.R.); julia.georgieva@postgrad.curtin.edu.au (J.G.); Angela.jacques@curtin.edu.au (A.J.); kevin.netto@curtin.edu.au (K.N.); 2 School of Science, Psychology and Sport, Federation University Australia, Ballarat 3350, Australia

**Keywords:** balance, brain function, concussion, sub-concussion, rugby, transcranial magnetic stimulation

## Abstract

Concussion is known to detrimentally affect brain health. Rugby tackles commonly occur with high collision force between tackler and ball carrier, and low impact head contact is not uncommon. Cognitive deficits following a bout of soccer ball heading has been attributed to the impact and termed sub-concussion. Although soccer ball heading studies provide evidence for acute effects of sub-concussion, it is unknown whether this phenomenon occurs following rugby tackles. This study investigates the acute effects of rugby tackles on brain function and balance in rugby players. Twenty-six volunteers were assigned to either the ball carrier (9), tackler (9) or control (8) group. Controls performed running without the tackle. Outcome measures included corticomotor function using transcranial magnetic brain stimulation (TMS) and balance was assessed by a series of tasks performed on a NeuroCom Balance Master before and immediately after a tackle training drill. Following the tackling bout, the cortical silent period (cSP) increased for the tacklers with no change for ball carrier and control groups, and no differences between groups for balance measures were observed. Lengthening of cSP observed in the tacklers following the bout has been reported in studies of concussion and may indicate long term detrimental effects.

## 1. Introduction

Head injury in sport has attracted attention among neuroscientists due to the relationship, identified in early research, between concussion and severe neurological impairments [1]. Rugby union is of interest, as it is amongst the world’s most played sports [2,3,4] and a defining feature of rugby union is the typical method of tackling. Due to its physical nature, with players usually tackling from the front while moving in the opposite direction to the ball carrier, rugby union has a high risk of collision-associated injury [3]. 

Concussion is the most common type of traumatic brain injury (TBI) in contact sport and is described, structurally, as a diffuse axonal injury [5,6]. The mechanism of injury can be attributed to direct forces, which act on the head and indirect forces acting on the body associated with an abrupt deceleration of the head. In rugby, indirect forces are the most common, since tackling usually occurs with the ball carrier and tackler running directly at one another [5,6]. The forces occurring in a tackle can cause movement of the brain within the skull where the brain may impact on the internal surface of the skull, towards the front (coup), and then at the back (contrecoup) causing cerebral contusions leading to concussion [7]. 

Following head trauma, concussion may be diagnosed through symptoms including headaches, poor balance and difficulties with vision [6,8,9,10]. After taking time out from sport following a head injury, players usually return to sport where a similar injury may occur. There is now a large body of evidence linking the cumulative effects of repeated concussions and long-term neurological impairments [2,6,11,12]. For instance, a fivefold increase in cognitive impairments was found in retired soccer players who sustained concussion or repeated concussions throughout their playing career [8]. Severe sequelae were found to be prevalent in professional boxers who sustained concussion, in fact 17% of these boxers developed dementia pugilistica, which consists of both motor and cognitive symptoms akin to those indicative of Parkinson’s disease [11,12]. 

Although concussion is linked to long-term neurological deficits, a gap exists in knowledge of the prevalence of sub-concussion, particularly in sport [11,13,14]. A sub-concussive impact can be defined as an event that causes a brain insult with insufficient force to produce clinical signs and symptoms that characterise a concussion [15]. Not unlike concussion events, sub-concussive impacts can cause deficits in cognition and alterations in postural control [15,16,17]. For instance, reduced performance in attention and memory were found in university rugby players post season, with only 3% experiencing a diagnosed concussion [18]. Similarly, declining neurocognitive and neurophysiological functions were found in high school American football players who never exhibited symptoms of concussion [19]. While this may be the result of under reporting of concussion, the cumulative effects of sub-concussive impacts are thought to account mainly for this difference [18,19]. 

Furthermore, a link between repetitive sub-concussions, across a sports career that may last decades, and long-term neurological disorders like dementia and Alzheimer’s disease is now being recognized [11,13,16,17,20,21,22,23]. Some of this research has been performed over the length of a single sporting season and therefore quantifies the accumulative rather than the acute effects of such impacts.

A study by Di Virgilio et al. [24] investigated the acute effects of sub-concussive impacts on cognitive function. The investigators used transcranial magnetic stimulation (TMS) to quantify the effects of sub-concussion through changes in brain function, by assessing corticomotor inhibition of the motor cortex (M1) [24]. Di Virgilio et al. [24] assessed effects of a single exposure of repetitive sub-concussive impacts, which involved “heading” of a soccer ball on measures of cortical function in soccer players and found increased intracortical inhibition among the players [24]. This indicates neurochemical changes related to primary motor cortex dysfunction. In a later study from the same group of researchers, increased intracortical inhibition, altered motor unit recruitment strategies and decreased memory performance were found among boxers following three 3 min sparring bouts. This effect was found among the boxers compared with a control group and test values returned to baseline within 24 h of the sparring bouts [25]. Transcranial magnetic stimulation has been used to assess long term neurophysiological changes in M1 due to degenerative diseases such as Alzheimer’s and Parkinson’s disease [26] as well as TBI [27], and researchers have considered that the impairments detected among volunteers in these studies may be similar to those detected following sub-concussion [8,24].

Any link between sub-concussive impacts and acute effects on brain function are of great significance and provide strong support for further study of the long-term effects of sub-concussion in humans to guide management of athletes in contact sports [15,19,24]. The aim of this study was to investigate the acute effects of impacts from a bout of rugby tackling on brain function using TMS and balance tests. We hypothesised that there would be an increase in short interval intracortical inhibition (SICI) and long interval intracortical inhibition (LICI) and lengthening of the cortical silent period (cSP) in the tackler group following the bout of tackling compared with the ball carrier and control groups. We further hypothesised that balance would be affected more among the tacklers than the ball carriers and the control group.

## 2. Materials and Methods

Twenty-six participants gave written consent to take part in this study, which was approved by the Human Research Ethics Committee at Curtin University (HR231/2015). Volunteers were assigned to one of three groups, ball carrier (BG), tackler (TG) or control (CG). Sixteen male and two female rugby players took part in tackle drills of the study while another eight volunteers (4 male) were allocated to the control group. Group characteristics are outlined in Table 1. Nine participants from the experimental groups had a history of concussion and had been fully cleared to continue playing by their general practitioner more than six months prior to participating in the study. All rugby players were recruited from state league. All volunteers were over the age of 18 years, played rugby for a minimum of 1 year at premier or reserve grade, and were current members of a local rugby club.

Participants completed a health screening questionnaire and a TMS exclusion criteria form and were excluded if they answered yes to any of the criteria. Participants were informed that they could withdraw from the study at any time, without giving any reason to the researchers. 

### 2.1. Procedure

All participants took part in baseline testing which included measures of neuromuscular function, using specialised brain stimulation protocols and balance measures explained in detail in Section 2.2 and Section 2.3 below. Following baseline testing, participants in the TG and BG groups took part in the main testing session, which consisted of a simulated match load of tackling in a motion analysis laboratory. Male participants were partnered with a player of a similar playing level and physical stature, then randomly assigned to a ball carrier or tackler. Since two female players volunteered for the study, they partnered with each other in the tackle bout. A total of 15 tackles were made in a 5 by 3 m grid to best simulate a normal 15-a-side rugby union tackling drill. In the starting position, the ball carrier and tackler faced each other from opposite ends of the testing area, approximately 2 m behind a grid marked on the floor. 

Each player was required to run at 3 metres per second entering the grid and their speed was calculated and monitored using Vicon motion capture system (Vicon VX, Oxford Metrics, Inc., Oxford, UK). Players practiced the run through in a brief familiarisation session and warm up prior to beginning the tackling bout. The ball carrier began with ball in hand and was instructed to attempt to get past the tackler approaching from the opposite end, but to stay within the boundaries of the 5 by 3 m grid. Each participant was instructed to perform their tasks as they would during a normal rugby practice drill. Each tackle was deemed complete if the ball carrier passed the tackler and reached the opposite side of the grid; the tackler successfully tackled the ball carrier to the ground; or the tackle came to a standstill for 3 s. Players were signaled to approach the tackle grid at the prescribed speed every minute, allowing each participant around 50 s break between each tackle to return to their starting position and reset for the next tackle. Following the conclusion of the tackling load, baseline testing was repeated. 

The CG did not take part in the tackling procedure. Rather, each participant completed a total of 15 shuttle runs. Each shuttle was a total of 9 m in length, with participants required to run at 3 m per second with a 1 min break between each shuttle. Following the shuttle runs, baseline tests were repeated which consisted of balance and TMS measures as explained below.

### 2.2. Motor Cortical Function

Transcranial magnetic stimulation (TMS) has been used to measure neuromuscular function in a variety of athletic populations including Australian rules football players, soccer players and American football players [8,12,14,24]. 

Motor evoked potentials (MEP) were elicited from the first dorsal interosseous (FDI) muscle of the non-dominant hand and measured using electromyography (EMG). Motor evoked potentials (MEP) were recorded using a 1902 Quad-system and SN: RM1184. 1401 Micro3, SN: M4508 and the CED Signal software package (Cambridge Electronic Designs, Cambridge, UK). Electrodes were placed on the belly of the FDI muscle approximately 2 cm apart with a ground electrode placed over the styloid process of the radius of the same hand. Two Magstim 200 stimulators connected to a standard figure-of-eight coil via a BiStim unit (Magstim, Whitland, UK), were used in these experiments. The coil was placed over the hotspot of the contralateral M1 and stimulated with supramaximal single pulses with a duration of 1 ms [8,28]. The point that elicited the largest MEP peak-to-peak was marked using a semi-permanent pen [8]. The coil was angled with the handle at approximately 45° posterior to the sagittal plane with a slight downward tilt [8]. The resting motor threshold (rMT) was established as MEP’s exceeding 50 microvolts (µV) in at least 3 of the 5 stimuli with the mean of the 5 also exceeding 50 µV. 

Short interval intracortical inhibition was assessed using an interstimulus interval (ISI) of 3 milliseconds (ms) and conditioning pulses of 70%, 80% and 90% of rMT and a test pulse of 1 millivolt (mV) [14,28]. Long interval intracortical inhibition was tested using a conditioning pulse of 1 mV followed by a test pulse of 1 mV with an ISI of 100 ms or 150 ms [29]. Cortical silent period was elicited with stimulus intensity at 1.2 times active motor threshold (aMT) while the participant performed an isometric abduction of the index finger of 10% of the maximal voluntary contraction [30].

### 2.3. Balance Testing 

Static and dynamic balance were tested using A NeuroCom Balance Master with a long plate (Natus medical Inc., Clackamas, OR, USA). Three tasks were performed, and each participant was given 3 trials for each task. Participants indicated when they were ready for each trial to commence and trials were deemed incomplete and were repeated if participants lost balance or were unable to maintain their position for the required time. The first task was the modified Clinical Test of Sensory Interaction on Balance (mCITSIB), which has been used in a wide variety of athlete populations including sprinters, jumpers and rugby players [31]. This test measures the ability to stand under conditions with reduced sensory cues and is a sensitive, reliable measure for testing the influence of visual, vestibular and somatosensory input on static balance [31]. Each participant was instructed to stand on a firm and then foam surface with their eyes open and then closed, 3 times for 12 s each [31]. The test consisted of eyes open on a firm surface (EOFIRM), eyes closed on a firm surface (ECFIRM), eyes open on a foam surface (EOFOAM) and eyes closed on a foam surface (ECFOAM). Excursion of the centre of gravity (COG) was then recorded.

The second task was the unilateral stance assessment (ULSA), which has been utilised in rugby academy players and has shown good to excellent test-retest reliability [32]. Participants were asked to stand on their right foot with their eyes open (REO) and then closed (REC) and then on their left foot with their eyes open (LEO) and then eyes closed (LEC) for 30 s in each trial. The amount of postural sway was recorded.

The final task was the limits of stability test (LOS) which is a reliable measure of functional stability in healthy populations [33]. The test measures the range in which a person can transfer their centre of mass within their base of support. Without lifting their feet, participants were asked to lean to 8 different directions to move a cursor to targets on the NeuroCom screen which were placed to their left, right, front and behind. Measurements included the displacement of centre of pressure (DCL), maximal excursion (MXE), endpoint excursion (EPE), movement velocity (MVL), directional control (DCL) and reaction time (RT) using the force plate from the NeuroCom Balance Master. An average from 3 trials performed for each of the tests were calculated and stored using the NeuroCom Balance Master. 

### 2.4. Statistical Analyses

Motor evoked potentials were delivered and recorded in groups of 12 stimuli by measuring the peak-to-peak amplitude and taking an average score of the 12 data points. Inhibition and facilitation measures were relative to test alone condition. Data from male and female participants were included in the experimental and control groups. Linear mixed effects models with random subject effects were used to analyse group changes over time in order to ascertain if there were any differences from pre to post between the tackler, ball carrier and control groups. Results are summarised as predicted marginal means and corresponding 95% CIs. Stata version 15 (StataCorp, College Station, TX, USA) was used for statistical analyses and *p*-values of <0.05 were considered significant.

## 3. Results

### 3.1. Effect of Rugby Tackling on Corticomotor Excitability and Inhibition

**Figure 1:**
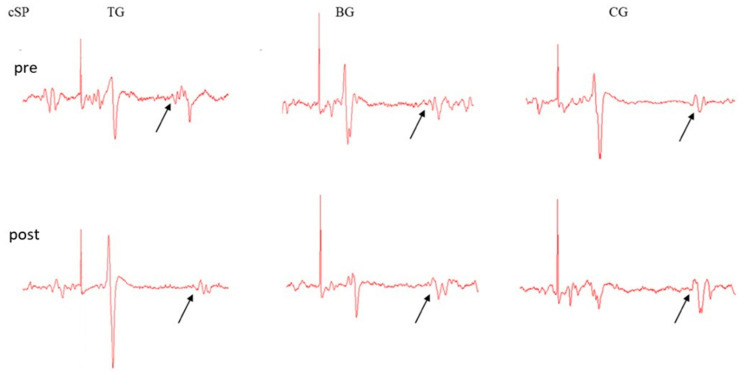
Raw data demonstrating the cortical silent period (cSP) lengthening of the cortical silent period from one participant pre and post tackle from the tackler group (TG), the ball carrier group (BG) and the control group (CG). The cSP was calculated from the test stimulus e artifact to the resumption of EMG activity indicated with an arrow.

**Figure 2:**
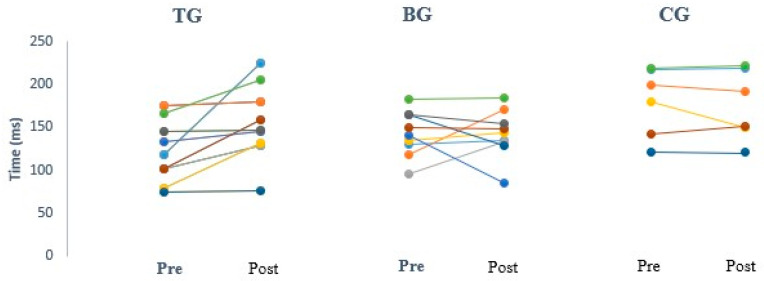
Change in cortical silent period in each participant pre and post intervention for the tackler (TG), ball carrier (BG) and control (CG) groups.

Overall, each participant in the BG and TG performed a total of 15 tackles. Immediately after the tackling session, there was a measurable increase in the primary outcome measure cSP for the TG, compared with baseline measurements and no change was found for the BG nor the CG. Examples of individuals from each group are shown in Figure 1. Pre and post data of cSP are shown for all participants in Figure 2. 

Figure 1 shows the cSP measures prior to and following the bout of tackling for the TG, BG and CG. While there was no change in cSP duration for the BG and CG, cSP for the TG increased from an average of 121.77 msec SEM milliseconds (msec) at baseline (95% Confidence interval (CI) = 97.94–145.61 msec) to 155.07 msec post tackle, (CI = 131.24–178.91) with a *p*-value of 0.001.

Intracortical inhibition was also assessed with no changes found in SICI at conditioning stimulus levels of 70% in all groups. There was a lifting off of SICI at a conditioning stimulus of 80% with MEP peak-to-peak values increasing from 0.81 mV to 1.56 mV (*p*-value 0.013) for the BG. SICI with a conditioning stimulus of 90% for the BG and CG showed a significant change (*p*-value 0.001 and 0.021). A significant change was also found for SICI with a conditioning stimulus of 90% when comparing the BG with the TG (*p* = 0.002). These data are summarized in Table 2 below.

### 3.2. Effects of Rugby Tackling on Balance

Table 3 highlights results from static and dynamic balance tests using A NeuroCom Balance Master. A significant decrease was found from baseline in the mCITSIB ECFOAM within the tackler (*p* = 0.05) and the control group (*p* = 0.001) post intervention while a significant difference (*p* = 0.03) was also found from baseline when comparing the tackling group against the ball carrier and control group post intervention. A significant decrease was found from baseline in the LOS Forward RT (*p* = 0.021), the LOS Back RT (*p* = 0.021), the LOS Left RT (*p* = 0.04) within the control group post intervention. A significant difference in the LOS Forward RT (*p* = 0.017) was also found when comparing the tackling group against the control group. 

A significant increase was found within the control group from baseline in the LOS Back MVL (*p* = 0.001), the LOS right MVL (*p* = 0.006) post intervention, while a significant increase was also found within the tackler group LOS Left MVL (*p* = 0.001) post intervention. A significant increase from baseline for the ball carrier group was found in LOS Forward EPE (*p* = 0.05), post intervention, while a significant increase was also found for the control group in the LOS Back EPE (*p* = 0.001) and the LOS Left EPE (0.004) post intervention. When comparing the ball carrier group with the tackler group from baseline to post intervention a significant difference in LOS Forward EPE (*p* = 0.041) was found, while a significant difference was also found when comparing the control group against the tackling group from baseline to post intervention in LOS Back EPE (*p* = 0.05). A significant increase was found within the control group from baseline in the LOS Left EPE (0.004), the LOS Back MXE (*p* = 0.025), post intervention. A significant difference was found from baseline when comparing the control group against the tackling group in the LOS Forward DCL (*p* = 0.048) post intervention.

## 4. Discussion

The aim of this study was to investigate the acute effects a simulated match load of tackling has on brain function and both static and dynamic balance. Following the tackling session, our data indicates changes in both outcome measures mentioned above, compared with baseline assessments. 

The main finding in this study was a significant increase in cSP after the bout of tackling for the tackling group while no change in cSP were detected for the control and ball carrier groups. This suggests transient changes in cortical function following sub-concussive, non-injurious clashes. These findings differ from most research on the topic, which proposes that changes in the cSP are due to concussion or the accumulative effects of concussion. For instance, De Beaumont et al. [8] reported a measurable increase in the cSP in American Football College athletes who sustained multiple concussions throughout their career compared with non-concussed athletes. 

Our results are in line with those of Di Virgilio et al. [24] who was the first to investigate sub-concussion acutely after a bout of soccer ball heading. The authors reported a significant increase in the cSP, while our findings are the first to do so in rugby players immediately after a typical match load of tackles. The increase in cSP indicates intracortical inhibition and is gamma-aminobutyric acid (GABA_B_) mediated, which serves as an inhibitor of the motor system [24]. The significance of this is of great relevance to sport as the same physiological process, that is an increase in cSP, is found in an array of neurological conditions. For instance, a lengthening of the cSP has been found in a cohort of patients with atypical Parkinsonian syndrome and Huntington’s disease [34]. In addition, a reduction in cSP has been found in patients with schizophrenia, depression and epilepsy [34], while the study by Pearce et al. [14] had a similar finding in a group of retired Australian Rules Football players who had experienced concussion compared with normal healthy controls. What is important to note is that these findings represent a chronic or lasting increase in the cSP, while our findings are an acute measure after a single bout of rugby tackling. Whether or not our findings contribute to a chronic or lasting effect in the cSP and to the long-term deficits mentioned above, remains unclear and further study is required. However, we have now established basis to investigate such a link.

Another outcome measure from this study demonstrates a measurable change in SICI for the tackler and ball carrier groups with a conditioning stimulus of 80% and 90% compared with baseline measures with no difference found in the control group. The amplitude of the MEP in the tackler group decreased significantly at 80 and 90% conditioning stimulus, while the ball carrier group had a significant increase in MEP amplitude showing a lifting off of inhibition. This may indicate motor system abnormalities and is likely related to GABA_A_ activity. GABA is the most common inhibitory neurotransmitter in the brain and excessive GABA_A_ activity is associated with memory impairment [35]. Numerous studies have found contrasting differences in SICI measures, with Trembley et al. [12] reporting no change in SICI measures comparing concussed with non-concussed athletes. Pearce et al. [14] reported a measurable increase in SICI with a conditioning stimulus of 80% when comparing Australian Rules Football players who had experienced a concussion with a control group who had not. These findings were in direct correlation with poorer outcomes in reaction times and fine motor control, and similar results were reported by De Beaumont et al. [8]. In the present study however, we found no correlation between SICI and performance in balance tasks. In fact, improvements in balance were found for both groups, the reason for which is unclear, but likely due to a practice effect [36,37]. We suggest the performance of tackling and of being tackled, may have had contrasting effects on intracortical inhibition due to differences in head movement during tackles. A recent study assessing likelihood of concussion in rugby union tacklers found that tacklers were significantly more likely to sustain a concussion when tackling from the front than from the side, and initial contact with the tacklers head or neck, as opposed to the shoulder, increased the likelihood of a concussion. The authors went further to explain that if the head position of the tackler just before contact was low, the likelihood of the player becoming concussed increased 4.67 times [38]. The position of the Tackler’s head, relative to the torso of the ball carrier was also an important contributing factor, increasing the likelihood of a concussion depending on the direction of the tackle [38]. This study concluded that skilled tackling technique is important for reducing the likelihood of head impact injuries and that avoidance movement of the ball carrier reduces the likelihood of concussion in the tackler [38].

As mentioned earlier, a significant improvement in balance was shown for both tackler and ball carrier groups in the various measures of the mCITSIB tasks although there was a great deal of variation across participant of all three groups (see Table 3 for details). A Study by Subasi et al. [37] reported that as little as a 10-min warm up can directly improve performance in balance measures such as mCITSIB and LOS, while Daneshjoo et al. [36] found that both static and dynamic balance was significantly improved in soccer players who performed a specific warm up regime involving running over a two-month period. We suspect a learning effect has taken place in the tackler, ball carrier and the control groups [36,37]. A sub-concussion does not produce clinical signs and symptoms that characterise a concussion, such as a loss of balance. Hence, it is unsurprising that no consistent changes across the balance outcome measures were found.

Di Virgilio [24] repeated baseline measurements on multiple occasions with a return in the cSP to baseline levels 24 h post intervention. This is perhaps a limitation to our research, as viewing the cSP 24 and 48 h post intervention would be a worthwhile endeavour on the lasting effects on corticomotor inhibition. However, the aim of the present study was to investigate measures that could be used to assess changes in cortical function following a series of sub-concussive events only since functional measures such as balance do not appear to be sensitive enough to assess sub-concussion. In this study we included a control group who performed the same amount of run throughs as the participants in the tackler and ball carrier groups to remove any influence of the physical activity of the protocol. However, a fourth group of control participants who performed no exercise would have added to the rigour of the study. Additionally, a larger cohort, with more female participants, would improve the study and likely address some of the statistical differences seen in some of the balance tests which may be due to chance with this number of participants. 

We suggest that future research now shift focus into the long-term effects of sub-concussion which has implications for contact sport as well as falls related injuries in older populations and other situations that may result in a head knock, including motor vehicle accidents. The finding that, like concussion, changes in the cSP can be detected in sub-concussion provides a basis for future research on this topic. It remains unclear as to the long-term effects of repeated non-injurious sub-concussion events. Whether or not this change is a protective mechanism against minor impacts such as those experienced in sports including rugby tackling is still unknown. Furthermore, whether these acute changes in cSP can contribute to lasting or chronic effects also remains unclear. 

## 5. Conclusions

In conclusion, this study found alterations in corticomotor function soon after a bout of rugby tackling. This is the first study to identify these changes in rugby players from non-injurious tackles. Although we found no deficits in balance, the changes in intracortical inhibition which indicate a change in brain function are of great interest, as similar findings can be observed following concussion. This research adds to the concerns by many that head knock events in sport or in other contexts are currently not well understood and that more research into this phenomenon is needed. 

## Figures and Tables

**Table 1:** Participant characteristics.

Group		Age	Height	Weight	Playing Experience	Number of Participants
Experimental	Males	23.43 ± 6.53	179.81 ± 7.06	91.55 ± 13.19	13.93 ± 4.84	16
	Females	18.5 ± 0.71	176.5 ± 7.78	79.5 ± 4.95	2 ± 0	2
Control	Males	32.8 ± 9.28	178.2 ± 12.36	84.2 ± 14.55	N/A	4
	Females	22.33 ± 4.04	164.67 ± 7.57	50.0 ± 5.29	N/A	4

Participant characteristics expressed as group mean ± *SD*.

**Table 2:** This table provides the mean scores for brain stimulation tests for groups before and after the tackle intervention with statistical interactions.

Outcome Measure	Group	Pre-Tackle			Post-Tackle	Pre-Post	*p* Interaction
		Mean	95%CI	Mean	95%CI		
cSP (ms)	TG	121.77	97.94–145.61	155.07	131.24–178.91	0.001 *	
	BG	141.85	116.57–167.13	141.73	116.44–167.01	0.990	0.022 *
	CG	170.78	143.75–197.81	164.63	137.61–191.66	0.587	0.009 *
1 mV	TG	1.96	1.07–2.86	1.28	0.35–2.22	0.173	
	BG	1.84	0.89–2.79	1.38	0.43–2.33	0.366	0.756
	CG	1.05	0.10–1.99	1.09	0.14–2.04	0.925	0.307
SICI 70% of rMT (mV)	TG	1.07	0.51–1.62	1.04	0.46–1.63	0.938	
	BG	0.50	−0.09–1.09	0.54	−0.05–1.13	0.892	0.88
	CG	0.28	−0.31–0.87	0.48	−0.11–1.07	0.533	0.618
SICI 80% of rMT (mV)	TG	1.60	1.09–2.12	0.85	0.31–1.40	0.012 *	
	BG	0.81	0.26–1.36	1.56	1.02–2.11	0.013 *	0.001 *
	CG	0.38	−0.17–0.93	0.62	0.07–1.17	0.430	0.021 *
SICI 90% of rMT (mV)	TG	2.47	1.58–3.36	1.60	0.67–2.52	0.039	
	BG	1.14	0.20–2.08	2.13	1.19–3.07	0.021	0.002 *
	CG	0.98	0.04–1.92	0.83	−0.11–1.77	0.720	0.232
LICI 100 Msec (mV)	TG	0.35	0.14–0.56	0.43	0.22–0.63	0.624	
	BG	0.26	0.05–0.47	0.13	−0.08–0.34	0.388	0.338
	CG	0.37	0.16–0.58	0.18	−0.03–0.39	0.201	0.211
LICI 150 Msec (mV)	TG	0.56	0.29–0.83	0.40	0.13–0.67	0.410	
	BG	0.26	−0.02–0.55	0.44	0.16–0.73	0.386	0.140
	CG	0.22	−0.07–0.50	0.27	−0.02–0.56	0.800	0.088

Pre-Post *p*-values—within group pre-post difference; *p* Interaction—*p*-values group × time interaction (carrier and control vs. tackler); cSP—cortical silent period; ms—milliseconds; mV—millivolts; SICI— short interval intracortical inhibition; rMT—resting motor threshold; LICI—long interval intracortical inhibition; SD—standard deviation (* *p* < 0.05).

**Table 3:** Static and dynamic balance. Pre- and post-tackle marginal means with within and between group pre-post differences.

Outcome Measure	Group	Pre-Tackle		Post-Tackle		Pre-Post	*p* Interaction
		Mean	95%CI	Mean	95%CI		
mCITSIB EOFIRM COG sway (°/s)	TG	0.33	0.23–0.43	0.36	0.25–0.46	0.563	
	BG	0.35	0.25–0.45	0.39	0.29–0.50	0.354	0.806
	CG	0.44	0.33–0.54	0.51	0.39–0.62	0.208	0.581
mCITSIB ECFIRM COG sway (°/s)	TG	0.34	0.27–0.42	0.39	0.32–0.47	0.160	
	BG	0.32	0.25–0.39	0.33	0.26–0.40	0.755	0.440
	CG	0.41	0.33–0.48	0.37	0.29–0.45	0.301	0.088
mCITSIB EOFOAM COG sway (°/s)	TG	1.06	0.66–1.45	0.59	0.20–0.98	0.098	
	BG	0.53	0.14–0.92	0.54	0.15–0.94	0.953	0.225
	CG	0.65	0.24–1.06	0.69	0.24–1.13	0.908	0.230
mCITSIB ECFOAM COG sway (°/s)	TG	1.19	0.99–1.40	1.12	0.92–1.33	0.269	
	BG	1.29	1.08–1.50	1.16	0.95–1.37	0.050	0.547
	CG	1.43	1.21–1.64	1.14	0.92–1.36	<0.001 *	0.030 *
ULSA LEO COG sway (°/s)	TG	0.74	0.07–1.42	0.76	0.08–1.44	0.973	
	BG	0.69	0.02–1.37	1.60	0.92–2.28	0.062	0.196
	CG	0.80	0.08–1.52	0.74	0.02–1.46	0.903	0.911
ULSA LEC COG sway (°/s)	TG	3.23	1.46–5.00	3.49	1.72–5.26	0.790	
	BG	3.87	2.10–5.64	4.24	2.47–6.01	0.710	0.941
	CG	2.13	0.25–4.00	3.45	1.57–5.33	0.212	0.469
ULSA REO COG sway (°/s)	TG	0.77	0.03–1.51	0.81	0.07–1.55	0.942	
	BG	1.11	0.37–1.85	1.62	0.88–2.36	0.345	0.537
	CG	0.80	0.01–1.59	0.85	0.06–1.64	0.930	0.989
ULSA REC COG sway (°/s)	TG	2.77	1.25–4.29	2.58	0.97–4.18	0.840	
	BG	4.01	2.49–5.53	2.58	1.06–4.10	0.116	0.346
	CG	3.91	2.30–5.53	2.56	0.95–4.18	0.160	0.388
LOS Forward RT (sec)	TG	0.59	0.45–0.72	0.67	0.54–0.81	0.300	
	BG	0.69	0.55–0.82	0.74	0.61–0.88	0.501	0.797
	CG	0.71	0.56–0.85	0.50	0.36–0.65	0.021	0.017 *
LOS Back RT (sec)	TG	0.53	0.23–0.82	0.51	0.21–0.81	0.942	
	BG	0.58	0.29–0.88	0.61	0.32–0.91	0.889	0.880
	CG	0.58	0.26–0.89	1.03	0.71–1.34	0.048 *	0.137
LOS Right RT (sec)	TG	0.63	0.51–0.76	0.48	0.36–0.61	0.020 *	
	BG	0.61	0.48–0.73	0.60	0.47–0.72	0.875	0.125
	CG	0.65	0.52–0.78	0.55	0.42–0.69	0.159	0.569
LOS Left RT (sec)	TG	0.53	0.44–0.62	0.53	0.44–0.62	0.880	
	BG	0.59	0.50–0.68	0.58	0.49–0.67	0.724	0.887
	CG	0.60	0.50–0.69	0.50	0.41–0.60	0.040 *	0.164
LOS Forward MVL (%)	TG	5.50	3.96–7.04	5.39	3.85–6.93	0.867	
	BG	4.84	3.31–6.38	5.76	4.22–7.29	0.170	0.276
	CG	5.38	3.75–7.00	6.58	4.95–8.20	0.088	0.176
LOS Back MVL (%)	TG	2.86	2.10–3.61	3.62	2.86–4.38	0.081	
	BG	3.04	2.29–3.80	3.72	2.96–4.48	0.123	0.886
	CG	2.66	1.86–3.47	4.19	3.38–4.99	0.001	0.236
LOS Right MVL (%)	TG	6.32	4.65–8.00	7.08	5.40–8.75	0.422	
	BG	5.97	4.29–7.64	4.63	2.96–6.31	0.157	0.116
	CG	5.29	3.51–7.07	8.03	6.25–9.80	0.006	0.148
LOS Left MVL (%)	TG	6.46	4.91–8.00	8.91	7.36–10.46	0.001	
	BG	5.84	4.30–7.39	6.96	5.41–8.50	0.139	0.206
	CG	7.88	6.23–9.52	8.31	6.67–9.95	0.538	0.065
LOS Forward EPE (%)	TG	86.67	76.42–96.92	86.44	76.19–96.69	0.959	
	BG	74.56	64.31–84.81	86.89	76.64–97.14	0.005 *	0.041 *
	CG	88.75	77.88–99.62	80.75	69.88–91.62	0.083	0.220
LOS Back EPE (%)	TG	50.11	41.25–58.97	47.89	39.03–56.75	0.598	
	BG	55.44	46.59–64.30	55.89	47.03–64.75	0.916	0.655
	CG	46.50	37.10–55.90	61.38	51.98–70.77	0.001 *	0.005 *
LOS Right EPE (%)	TG	85.33	78.04–92.62	93.56	86.26–100.85	0.058	
	BG	83.11	75.82–90.40	88.78	81.49–96.07	0.191	0.677
	CG	85.00	77.27–92.73	89.63	81.89–97.36	0.314	0.569
LOS Left EPE (%)	TG	96.22	87.38–105.06	93.89	85.05–102.73	0.585	
	BG	88.22	79.38–97.06	90.44	81.61–99.28	0.603	0.451
	CG	95.13	85.75–104.50	104.25	94.88–113.62	0.044 *	0.066
LOS Forward MXE (%)	TG	97.33	89.23–105.43	94.22	86.12–102.32	0.343	
	BG	91.89	83.79–99.99	97.56	89.46–105.66	0.084	0.058
	CG	95.13	86.53–103.72	91.88	83.28–100.47	0.350	0.977
LOS Back MXE (%)	TG	61.00	51.12–70.88	61.00	51.12–70.88	1.000	
	BG	68.89	59.01–78.77	69.89	60.01–79.77	0.866	0.905
	CG	57.75	47.27–68.23	71.88	61.40–82.35	0.025 *	0.102
LOS Right MXE (%)	TG	105.11	98.26–111.96	103.78	96.93–110.62	0.717	
	BG	102.00	95.15–108.85	98.33	91.49–105.18	0.318	0.653
	CG	98.38	91.11–105.64	103.13	95.86–110.39	0.223	0.256
LOS Left MXE (%)	TG	105.22	97.24–113.20	103.44	95.47–111.42	0.706	
	BG	100.67	92.69–108.65	97.78	89.80–105.76	0.539	0.867
	CG	108.50	100.04–116.96	111.13	102.66–119.59	0.599	0.521
LOS Forward DCL (%)	TG	85.89	82.79–88.99	87.56	84.45–90.66	0.341	
	BG	89.00	85.90–92.10	87.89	84.79–90.99	0.526	0.262
	CG	87.63	84.33–90.92	84.25	80.96–87.54	0.069	0.048 *
LOS Back DCL (%)	TG	66.22	58.65–73.79	65.89	58.32–73.46	0.950	
	BG	75.78	68.21–83.35	71.11	63.54–78.68	0.379	0.563
	CG	64.88	56.85–72.90	69.88	61.85–77.90	0.374	0.490
LOS Right DCL (%)	TG	75.22	70.88–79.56	77.33	72.99–81.67	0.398	
	BG	81.89	77.55–86.23	79.44	75.10–83.79	0.328	0.198
	CG	78.38	73.77–82.98	77.13	72.52–81.73	0.637	0.356
LOS Left DCL (%)	TG	73.89	69.85–77.93	77.11	73.07–81.15	0.084	
	BG	80.56	76.52–84.59	79.33	75.29–83.37	0.512	0.092
	CG	78.50	74.22–82.78	80.63	76.34–84.91	0.282	0.686

Pre-Post, *p*-values within group pre-post difference; *p* Interaction, *p*-values group/time interaction (ball carrier vs. control vs. tackler); mCITSIB, modified clinical test of sensory interaction in balance; ULSA, unilateral stance assessment; COG, centre of gravity; LOS, limits of stability, 95% confidence interval (* *p* < 0.05).
